# The Effects of *Porphyromonas gingivalis* on Atherosclerosis-Related Cells

**DOI:** 10.3389/fimmu.2021.766560

**Published:** 2021-12-23

**Authors:** Jiaqi Zhang, Mengru Xie, Xiaofei Huang, Guangjin Chen, Ying Yin, Xiaofeng Lu, Guangxia Feng, Ran Yu, Lili Chen

**Affiliations:** ^1^ Department of Stomatology, Union Hospital, Tongji Medical College, Huazhong University of Science and Technology, Wuhan, China; ^2^ School of Stomatology, Tongji Medical College, Huazhong University of Science and Technology, Wuhan, China; ^3^ Hubei Province Key Laboratory of Oral and Maxillofacial Development and Regeneration, Wuhan, China

**Keywords:** *Porphyromonas gingivalis*, atherosclerosis, endothelial dysfunction, foam cell, T cell

## Abstract

Atherosclerosis (AS), one of the most common types of cardiovascular disease, has initially been attributed to the accumulation of fats and fibrous materials. However, more and more researchers regarded it as a chronic inflammatory disease nowadays. Infective disease, such as periodontitis, is related to the risk of atherosclerosis. *Porphyromonas gingivalis* (*P. gingivalis*), one of the most common bacteria in stomatology, is usually discovered in atherosclerotic plaque in patients. Furthermore, it was reported that *P. gingivalis* can promote the progression of atherosclerosis. Elucidating the underlying mechanisms of *P. gingivalis* in atherosclerosis attracted attention, which is thought to be crucial to the therapy of atherosclerosis. Nevertheless, the pathogenesis of atherosclerosis is much complicated, and many kinds of cells participate in it. By summarizing existing studies, we find that *P. gingivalis* can influence the function of many cells in atherosclerosis. It can induce the dysfunction of endothelium, promote the formation of foam cells as well as the proliferation and calcification of vascular smooth muscle cells, and lead to the imbalance of regulatory T cells (Tregs) and T helper (Th) cells, ultimately promoting the occurrence and development of atherosclerosis. This article summarizes the specific mechanism of atherosclerosis caused by *P. gingivalis*. It sorts out the interaction between *P. gingivalis* and AS-related cells, which provides a new perspective for us to prevent or slow down the occurrence and development of AS by inhibiting periodontal pathogens.

## Introduction

Cardiovascular disease (CVD) is the most common cause of death worldwide, which leads to about 16.7 million people losing their lives each year (1). Atherosclerosis (AS), a chronic disease that often occurs in large- and medium-sized arteries, is regarded as the pathogenetic basis of most CVDs (2). Although traditional risk factors for AS, such as hyperlipidemia, hypertension, and smoking, have been effectively reduced, the incidence of atherosclerotic diseases remains high (3). In the past few decades, new evidence that AS is a chronic inflammatory disease emerged (4). A variety of pathogens, such as *Chlamydia pneumoniae* (*C. pneumoniae*) (5), *P. gingivalis* (6), and *Helicobacter pylori* (7), have been detected in human AS plaque lesions and promote the progression of AS, which suggests that pathogen infection may participate in the formation of AS plaques (8).

Periodontitis, which affects 11.2% of the population worldwide, is the sixth most common disease and a highly and prevalently chronic non-communicable disease (9). Many epidemiological and clinical studies have shown that periodontal disease is related to carotid AS (10, 11). People suffering from periodontitis have a higher risk of AS/CVD, and its risk ratio ranges from 1.074 to 1.213, 95% CI (12–15). However, few studies suggested that the link between these two diseases is not very clear (16). Maybe the clinical association between periodontal disease and AS was unsure, but numerous animal experiments have confirmed the promotion role of periodontal pathogens in the progress of AS (17, 18). Periodontal pathogens, such as *P. gingivalis* (19), *Aggregatibacter actinomycetemcomitans* (20), and *Tannerella forsythia* (21), have been detected in human AS plaque lesions. Among the periodontal bacteria detected, the detection rate of *P. gingivalis* is particularly high (22–24), and studies about the promotion effect of *P. gingivalis* on AS are also the most common. It seems that out of oral or periodontal pathogens, *P. gingivalis* has the advantage in AS pathogenicity.

*P. gingivalis* is the main component of the subgingival plaque in patients with chronic periodontitis. It is not only involved in inflammation and tissue destruction during periodontal disease (25) but also related to the inflammatory pathology of distal body organs, including AS and Alzheimer’s disease (AD) (26, 27). It can enter the blood system through ulcers in the epithelium and lymphatic vessels after treatment intervention (subgingival scaling, surgical periodontal therapy) or daily activities (brushing, chewing), and then survive and colonize in other organs (28). *P. gingivalis* (6) and its contents, such as fimbriae (29) and DNA (30), have been detected in human atherosclerotic plaques. In recent years, a lot of studies proved that *P. gingivalis* could accelerate atherosclerosis (18, 25, 31–35); the underlying mechanisms have also been discussed. Most studies and reviews focus on endothelial cells (ECs), but there are many other kinds of cells involved in the development of AS, like vascular smooth muscle cells (VSMCs), macrophages, and T cells. *P. gingivalis* can also affect the functions of these cells. In order to give a more systematical and comprehensive understanding of the promoting role of *P. gingivalis* on AS, here we summarize the effects and internal mechanisms of *P. gingivalis* on all types of cells related to AS.

## Characteristics of *P. gingivalis*

*P. gingivalis* is an obligate asaccharolytic gram-negative bacteria. It is the most dominant bacteria in periodontitis and has been proven as the main pathogenic bacteria in patients with chronic periodontitis (36, 37). Researchers have clarified that *P. gingivalis* can promote the development and aggravation of systemic diseases, such as cardiovascular diseases, largely because of its ability to modulate the entire ecosystem by changing the immune response of the host to survive and persist in host tissues (38), which is related to interacting with various host receptors and changing the inflammation and complement system signal transduction pathways as well as cell cycle and apoptosis (39). Gingipains (40) and outer membrane vesicles (OMVs) (41) secreted by *P. gingivalis*, with its lipopolysaccharides (LPS) (42), proteins (43), and fimbriae (44), make *P. gingivalis* highly pathogenic and thus persistent in host tissues and promote the emergence of dysbiosis.

**Figure 1:**
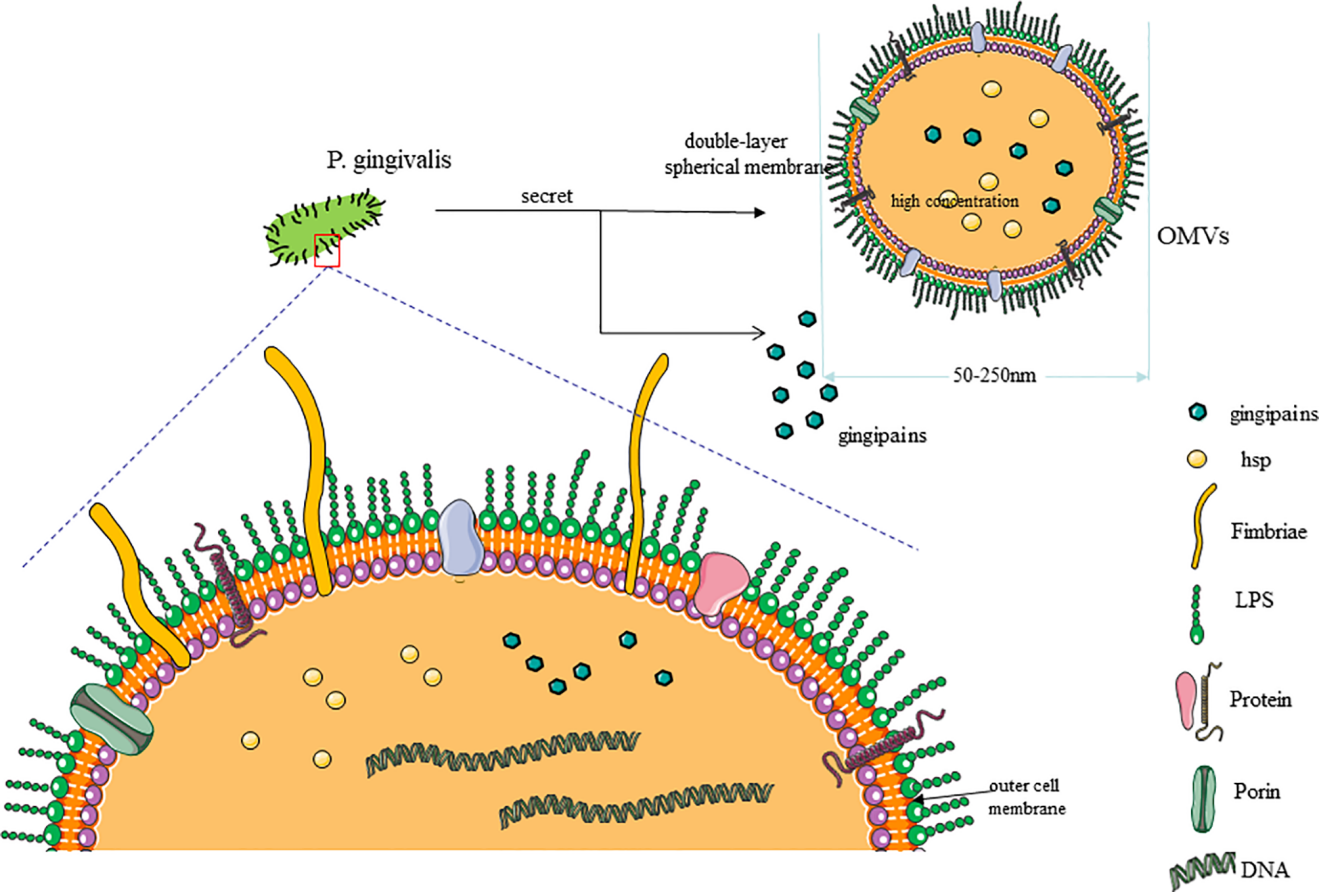
Characteristic of *P. gingivalis*. (1) As the most common periodontal pathogen, *P. gingivalis* is composed of cell membrane and genetic material. The outer layer of the cell membrane has a large number of fimbriae, proteins, and channels. (2) The pathogenicity of *P. gingivalis* mainly comes from its own structural components (lipopolysaccharide, fimbriae, and heat shock proteins) and secretory components (gingipains and outer membrane vesicles, OMVs). (3) OMVs have a double-layer spherical membrane and contain a lot of pathogenic factors with high concentration.

LPS is an important part of the outer layer of *P. gingivalis* and has a strong pathogenic effect (37). It can induce toll-like receptor (TLR)-specific immune upregulation, in which TLR4 and TLR2 are the main receptors (33, 45), so as to trigger inflammation and immune responses between the host through TLRs. *P. gingivalis* fimbriae, comprised of FimA and Mfa1 subunits, is a crucial factor in the interaction between bacteria and host tissues, promoting the adhesion and invasion of bacteria to target sites (46). It also can be recognized by TLRs on ECs (22), macrophages (47), and immune cells (48), thereby activating the cells to produce cytokines and adhesion molecules. Experiments showed that infection with the fimbriae-deficient mutant DPG3 of *P. gingivalis* had a minimal effect on pro-AS (49, 50). The heat shock protein 60 (HSP60) of *P. gingivalis* is remarkably immunogenic (51), and existing reports indicate that the HSP60 IgG antibody titers in patients with AS and periodontitis are elevated (52). As the main secretory component of *P. gingivalis*, gingipains consist of arginine-gingipain (Rgp) and lysine-gingipain with hemagglutinin (Hag)-adhesin domain, with 85% of extracellular proteolytic ability coming from it (53), providing *P. gingivalis* the ability of tissue destruction, and can modulate the expression of cytokines and immunoglobulins and thus affect the immune responses of the host cells (54). With research going on, the OMVs with double-layer, spherical, membrane-like structures secreted by *P. gingivalis* have been proven, with a size of about 50–250 nm (55), to contain LPS, outer membrane proteins, phospholipids, and DNA inside (56). The OMVs make a large number of pathogenic factors highly concentrated and avoid the degradation and destruction of proteolytic enzymes, thus greatly improving the toxicity from *P. gingivalis* (41) (**Figure 1**).

There are some perspectives from new studies that the vasculature can be invaded by *P. gingivalis via* an ulcerative epithelium (57) and lymphatic vessels (58); then, *P. gingivalis* could be internalized in gingival epithelial cells and KB cell lines with ECs through the “folding” mechanism which caused severe folds of the host cell membrane at the invasion site and was internalized in the form of spacious vacuoles (59). In addition, studies have shown that *P. gingivalis* can transmit among different types of cells in vascular tissues (60). All the properties described above support that *P. gingivalis* invades distant tissues and colonize in parts other than the oral cavity, leading to a more serious outcome of the systemic disease (**Figure 1**).

## Pathogenesis of Atherosclerosis

**Figure 2:**
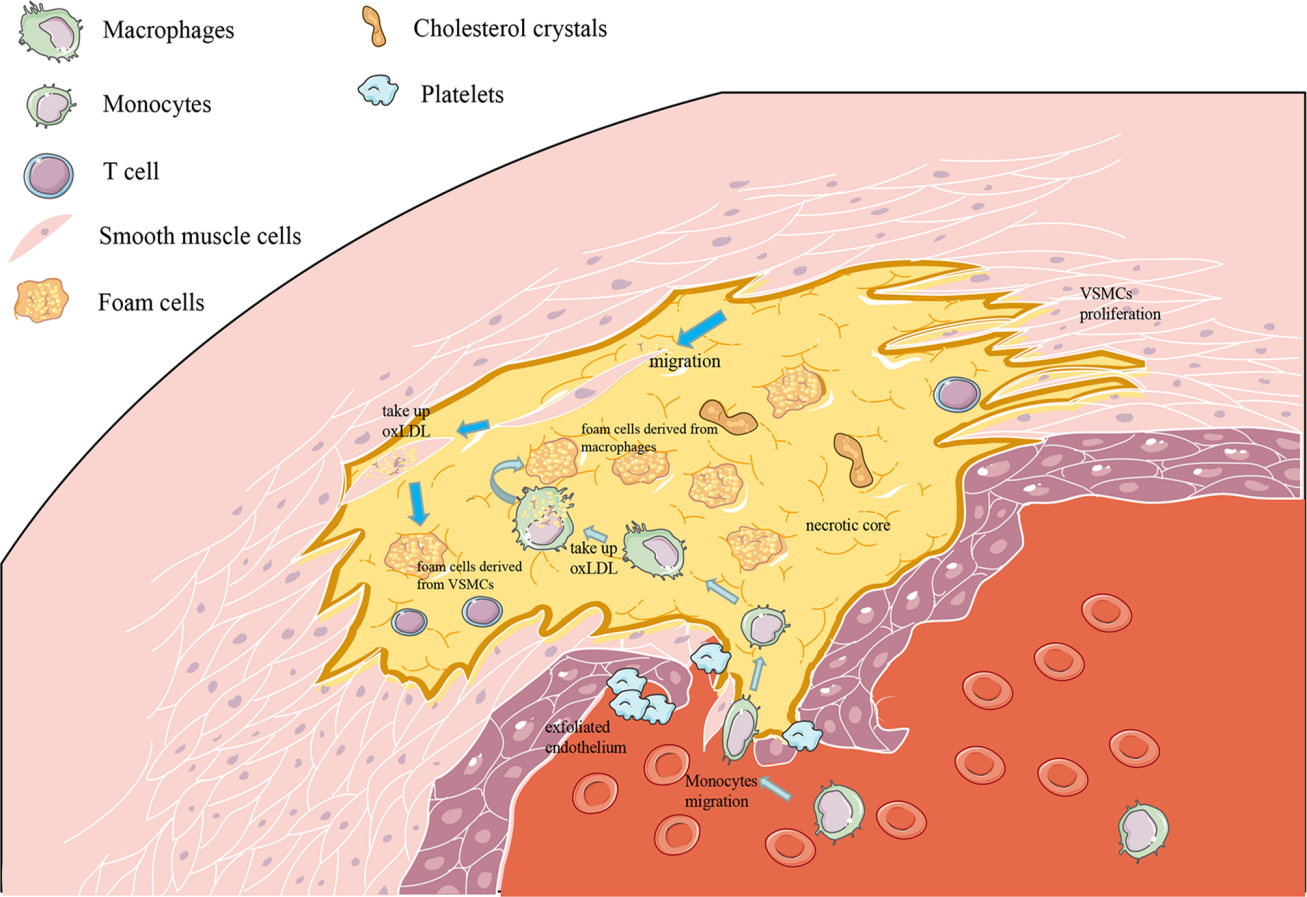
Pathogenesis of atherosclerosis (AS). (1) As the picture shows, AS occurs in the intima, where endothelial cell damage and monocyte migration and adhesion occur. (2) After the monocytes enter the inner membrane, they differentiate into macrophages and increase the uptake of oxLDL to become foam cells. (3) Outside the intima, the media contains vascular smooth muscle cells, which proliferate and migrate to the intima and then differentiate into vascular smooth muscle cell-derived foam cells.

AS is a continuous course of decades, along with the accumulation of fatty material and plaque formation in the innermost lining of the artery, causing acute coronary syndromes, myocardial infarction, or stroke (16). The pathological process of atherosclerosis is related to the physiological activities and transformation of various cells, including ECs, VSMCs, macrophages, T cells and, dendritic cells (DCs). At the onset, in some atherosclerotic lesions, the vascular endothelium will be abnormally stimulated. As the disease progresses, there will be shed areas in the endothelium, and platelets stick to exposed areas (43, 44). Subsequently, circulating monocytes are recruited from the blood to the subintima; they internalize and modify lipoproteins and finally differentiate into foam cells (49). VSMCs proliferate, migrate, and produce a sizeable extracellular matrix (ECM), which is the main component of the fibrous cap of AS plaques. In addition, VSMCs can also internalize lipids and differentiate into foam cells (50). Last but not least, the immune response caused by T cells and DCs also plays an indispensable role in the pathological development of AS (**Figure 2**).

### ECs in AS

The endothelium, as the outermost layer between blood and arterial intima, is the initial area of atherosclerotic lesions (61). It is a key factor in regulating vascular homeostasis because of the barrier function of ECs and the ability to regulate the phenotypes of the vascular wall (62). In the prone areas of atherosclerotic lesions, the vascular endothelium firstly leaks, activates, and malfunctions after being stimulated by dyslipidemia, hypertension, or pro-inflammatory mediators, which is also called endothelial dysfunction (63, 64). When the endothelium leaks, the permeability of the endothelium is destroyed, and more circulating low-density lipoprotein (LDL) enters (61). Meanwhile, oxidative stress occurs in the endothelium and produces a lot of superoxide. LDL accumulates in the vascular intima and is oxidized by this superoxide, and then being oxidized low-density lipoprotein (oxLDL), it can induce and bind to cell surface adhesion molecules to activate ECs or be recognized by T cells and drive an autoimmune response (65, 66). As the disease progresses, exfoliated areas appear in the endothelium, and platelets may be adhering to this exposed subendothelial tissue. Before the morphological changes of AS occurred, the endothelial function changed (67). It is a complex pathophysiological event, including endothelial activation, impaired vascular tone, and other endothelial phenotypic changes (61). The pro-inflammatory and procoagulant state of ECs is called endothelial activation (68). In this state, ECs express many chemokines, cytokines, and adhesion molecules, which trigger leukocytes to homing, adhering, and migrating to target tissues. The activated ECs first selectively recruit circulating monocytes from the blood to under the inner membrane, where the monocytes differentiate into macrophages, modify lipoproteins, internalize a large number of lipids, and finally differentiate into foam cells (this is a sign of early fatty streak disease) (69). ECs can also produce perlecan and heparan sulfate proteoglycans under mechanical forces, which are intimately involved in the endothelial inhibition of VSMC proliferation (70). The outer edge of the plaque is rich in inflammatory cells, which further regulates the pro-inflammatory phenotype of ECs and ultimately leads to the instability of the plaque structure (71). ECs can acquire myofibroblast-like properties through endothelial cell–mesenchymal transition (EndMT), which is involved in the occurrence of AS (72).

### Vascular Smooth Muscle Cells in AS

VSMCs are the main cell types that exist in various stages of atherosclerotic plaques and can differentiate into various cell phenotypes, including macrophages and foam cells (73). The response of VSMCs to arterial injury and lipid infiltration is the main pathological process of atherosclerotic plaque development (74). VSMCs are the primary source of elastin and interstitial collagen in the inner membrane and a vital part of the ECM, which allows the artery to be compliant and elastically retractable (75, 76). In the pre-AS stage, the proliferation and migration of VSMCs contribute to its migration to the inner membrane from mid-arterial (77). VSMCs secrete a large amount of ECM, which promotes diffuse intimal thickening of the vessel wall (78, 79). In the early stage of AS, VSMCs proliferate, absorb oxLDL, and form foam cells, which promotes pathological intimal thickening (PIT). The arterial intima forms a deep pool of extracellular lipids, and a large number of VSMCs and ECMs accumulate. Microcalcifications (0.5–15-μm spots) are often produced in the lipid pool of PIT, usually near the boundary of the medium, which may be the result of VSMC apoptosis (80–83). At the same time, VSMCs are an essential source of macrophages in AS, and macrophages derived from VSMCs participate in the process from PIT to fibrotic plaques. Finally, the fibrous cap of the AS plaque is formed (84). In the advanced stage of AS, after the death or apoptosis of VSMCs, the accumulated lipids are released outside the cell, forming a necrotic core in the plaque and, at the same time, acting as an antigen to activate T cells to secrete inflammatory mediators, resulting in unstable plaque formation (85).

### Macrophages in AS

Macrophages are the main immune cells in atherosclerotic lesions (86). Macrophages are critical and a requisite in every stage of AS (87), from its initiation and expansion to the rupture caused by necrosis and clinical manifestations and to the regression of lesions. The blood monocytes are primarily derived from focal macrophages (88), and circulating monocytes enter the arterial hemodynamic stress site by adhering to the ECs of the susceptible artery lumen (89). The different phenotypes of macrophages enable them to perform different functions (90). Circulating monocytes in the blood bind to adhesion molecules, monocyte chemoattractant protein-1 (MCP-1), and intercellular adhesion molecule-1 (ICAM-1) expressed in activated ECs (91). Then, they enter the plaque through three activities: capture, rolling, and migration. Each step is regulated by multiple molecular factors, sometimes overlapping (89). Once monocytes enter the inner membrane, they can differentiate and mature into macrophages and acquire characteristics associated with repairing or less pro-inflammatory monocyte/macrophage populations (92). The formation of foam cells by macrophages is an important process of pathological changes in AS, and it is also one of the main sources of foam cells in the lipid pool (87). Macrophages increase the uptake of oxLDL while reducing cholesterol efflux, which, in turn, leads to the deposition of intracellular esterified cholesterol and the production of foam cells derived from macrophages. Inflammatory macrophages secrete cytokines and proteases, increasing the expansion of diseased cells, causing changes in plaque morphology, and leading to plaque rupture and acute intraluminal thrombosis. In contrast, dissociated macrophages perform functions related to stabilizing plaques, including removing dead cells (exocytosis) to stabilize plaques and secreting collagen to form protective scars on the lesions (93).

### T Cells in AS

The latest research shows that AS is a chronic inflammatory disease (86). Tregs and effector T cells mainly control the adaptive immune process of AS (92). For plaques in AS, various T cell lineages are crucial for their initiation, progression, and stability (94). T helper cells 1 (TH1) can accelerate atherosclerosis, and Tregs can inhibit the progression of atherosclerosis (66). It should be noted that Tregs can become pro-atherogenic cells. The complexity of TH1 and Tregs functions is due to activating or inhibiting the roles of other T cells, promoting the production of high-affinity resistance and cytotoxicity (95). The roles of other T cell subgroups like CD8+ T cells and γδ T cells and TH cell subgroups such as TH2 and TH9 are not much understood (66).

### Dendritic Cells in AS

When monocytes are recruited to enter the subendothelial layer in the early stage of AS lesions, another type of immune cell, DCs, also takes the opportunity to invade the subendothelial intima preferentially, forming a structure like the cutaneous Langerhans cell network (96). The role of DCs in the pre-AS stage is a double-edged sword. It can have a protective effect by reducing effector T cell proliferation and inhibiting IFN-γ production (97). On the other side, DCs under the inner membrane has a pro-atherosclerotic effect. It can ingest cholesterol to promote lipid accumulation and foam-like lesions (98). Treg homeostasis can be regulated by DC-derived chemokines, which suggests that DCs control T cell responses by multifarious mechanisms to achieve anti- or pro-inflammatory effects in AS (99). Last but not least, DCs can infiltrate arterial walls, which may destabilize atherosclerotic plaques and contribute to inflammatory development in AS (100) (**Figure 2**).

## The Influence of *P. gingivalis* on ECs

**Figure 3:**
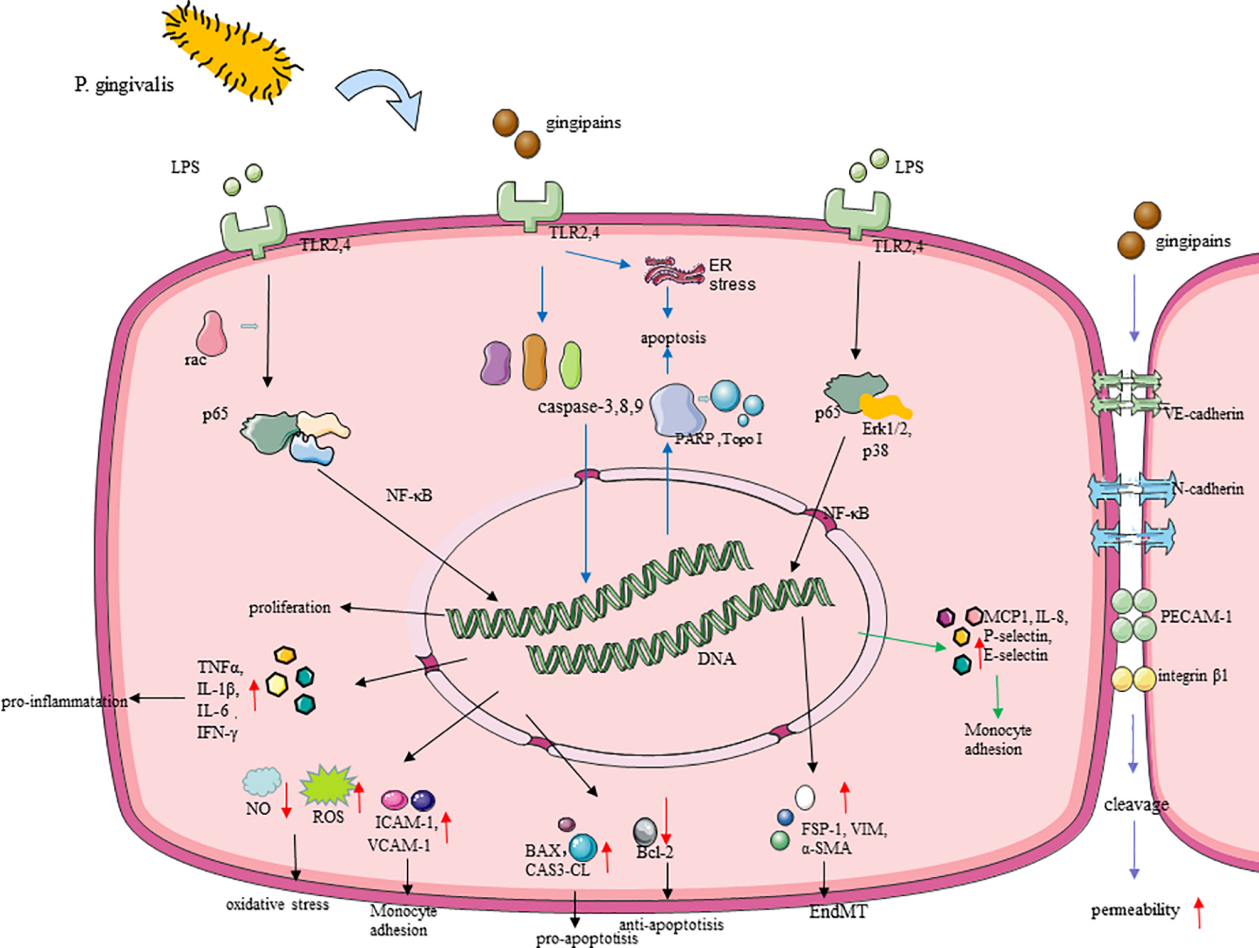
Molecular cascades activated by *P. gingivalis* in endothelial cells (ECs). (1) TLR is believed to mediate the recognition of *P. gingivalis*. *P. gingivalis* promotes EC oxidative stress through the TLRs–NF-κB signal axis and NLRP3 inflammasomes. *P. gingivalis* leads to nitrifying stress and impaired endothelial function, with upregulating iNOS, downregulating eNOS, and regulating the release of NO in EC (86), which is associated with the change of the GSK-3β/BH4/eNOS/Nrf2 pathway. (2) *P. gingivalis* gingipains induced endothelial cell (EC) apoptosis by activating caspase-3,8,9, and its gingipains can also induce EC apoptosis mainly through inducing the cleavage of PARP and Topo I. (3) *P. gingivalis* lipopolysaccharide promoted EndMT through the regulation of p38, Erk1/2, and p65. (4) *P. gingivalis* enhanced monocyte migration with an increased expression of MCP-1, ICAM-1, IL-8, P-selectin, and E-selectin in ECs. (5) Gingipain destroyed the endothelial cell–cell junction through inducing the cleavage of VE-cadherin, N-cadherin, PECAM-1, and integrin β1.

In the past few years, research has proposed that *P. gingivalis* has the capability to act and invade ECs, induce endothelial dysfunction, destroy endothelial integrity, and then promote the formation and development of atherosclerotic plaques (101) (**Figure 3**).

### *P. gingivalis* Activates Endothelial Oxidative Stress and Promotes Inflammation Response

Oxidative stress is fundamental to AS. Endothelial oxidative stress promotes the adhesion of monocytes and the release of pro-inflammatory cytokines, leading to endothelial dysfunction, which is a precursor to atherosclerotic lesions (61). Recent studies have proved that *P. gingivalis* induces severe endothelial oxidative stress (102). *P. gingivalis* can significantly increase the output of total reactive oxygen species (ROS) and superoxide free radicals *in vitro*, destroying endothelial function. This process is mainly promoted by the TLRs–NF-κB signal axis (22). TLRs mediate the recognition of *P. gingivalis* LPS and then activate the downstream signaling pathway NF-κB and its active subunit p65, thereby triggering subsequent oxidative stress (22), and peroxisome proliferator-activated receptor may be also involved in modulating oxidative stress during this process (103). Moreover, *P. gingivalis* activated nucleotide-binding domain leucine-rich repeat (NLR) and promote the production of pyrin domain-containing receptor 3 (NLRP3) inflammasomes in ECs, which depends on ROS and LPS. Then, interleukin-1β (IL-1β) and IL-18 begin to be secreted, thereby promoting further inflammatory processes and oxidative stress in the endothelium (104). It is worth noting that, in the process of *P. gingivalis* promoting oxidative stress, there is also the influence of circadian clock disruption (22), which can provide new insights on the treatment of *P gingivalis*-accelerated atherosclerosis. By the way, nitric oxide plays an important role in maintaining homeostasis and anti-oxidative stress through inhibiting the production of ROS (105), and the action of *P. gingivalis* eventually leads to nitrifying stress and impaired endothelial function, which is achieved by upregulating inducible nitric oxide synthase (iNOS), downregulating endothelial nitric oxide synthase (eNOS), and regulating the release of NO in EC (106), with the change of glycogen synthase kinase-3 (GSK-3β)/tetrahydrobiopterin (BH4)/eNOS/nuclear factor erythroid-derived 2-like 2 (Nrf2) pathways (107). Animal experiments have shown that *P gingivalis* infection leads to a significant decrease in the bioavailability of BH4, which may be due to the inhibition of the expression of dihydrofolatereductase (DHFR) that predominates the conversion of BH2 to BH4 and the rate-limiting enzyme GCH-1 (GTP cyclohydrolase 1) responsible for the biosynthesis of BH4 (108). NrF2 can protect cells from oxidation by activating antioxidant enzymes, including GSH synthase (GCSc, GCSm) and heme oxygenase-1, which is essential for cell protection. In the vascular tissues of mice infected with *P gingivalis*, the level of NrF2 was significantly reduced (107). Interestingly, after the intervention of HAECs cells with *P. gingivalis*, the expression of DHFR was significantly inhibited, but the expression of DHFR did not change significantly *in vivo*. This may be due to the latter being a variety of periodontal pathogen infections after intervention.

After oxidative stress, *P. gingivalis* triggers the inflammatory response in vasculature. IL-1β, IL-6, TNFα, and interferon-gamma (IFN-γ), as pro-inflammatory factors, were increased by *P. gingivalis* in ECs (109, 110). It is also reported that *P. gingivalis* LPS-, fimbriae-, OMV-, and gingipain-stimulated ECs expressed high levels of MCP-1, ICAM-1, IL-8, P-selectin, and E-selectin, as well as their receptors C-C chemokine receptor type 2 and integrin αMβ2, of which all of them enhance monocyte migration and adhesion (50), thereby initiating and promoting inflammation and promoting the development of AS (111, 112). Multiple signaling pathways, including p38, c-Jun N-kinase, NF-κB, and activator protein 1 (AP-1), are involved in this process (113–116). What is more, the inner-out signal transduction of *P. gingivalis* fimbriae is mediated by Ras-related C3 botulinum toxin substrate 1 (Rac1) and phosphatidylinositol 3-kinase (PI3K) (117). In addition, with *P. gingivalis*-LPS stimulation in ECs, it secreted anti- chemotaxis and anti-adhesion proteins like Growth arrest-specific 6 (Gas6) and pro-adhesion proteins like ICAM-1 and macrophage migration inhibitory factor (MIF), and these signs of progress can be regulated by LncRNA GAS6-AS2 (118). MIF binds with the MHC class II invariant chain, called Ii/CXC motif chemokine receptor type 4; a matchable receptor complex, it facilitated monocyte adhesion, too (118).

### *P. gingivalis* Destroys the Endothelial Barrier

The permeability of the endothelial barrier is also a part of the inflammatory responses in the development of AS (119), which has been proven to be promoted by *P. gingivalis* (120). *P. gingivalis* and its gingipains, LPS, and OMVs may contribute to endothelial barrier destruction at the endothelial cell–cell junction by inducing the decomposition of adhesion molecules like VE-cadherin and N-cadherin. During this process, platelet endothelial cell adhesion molecule-1 (PECAM-1) and integrin beta1 were also cleaved and destroyed by *P. gingivalis* in ECs (120–123), thus allowing leukocyte transmigration in this system. *P. gingivalis* LPS can promote the internalization of VE-cadherin in ECs, which play an essential role in regulating EC permeability (123). Evidence have indicated that IL-8 directly increased endothelial permeability (124), and it can be upregulated by LPS of *P. gingivalis* with the activation of the NF-κB pathway (123). *P. gingivalis* also promoted vascular coagulation and inflammation which were mainly related to the degradation and inactivation of glycoprotein thrombomodulin on the surface of ECs (120, 125). The permeability of the endothelium increases, allowing bacteria to pass through the endothelium through loose connections. This may explain why *P. gingivalis* invading ECs is accompanied by a mixed infection of other periodontal pathogens.

*P. gingivalis* was able to induce ECs apoptosis and EndMT as well as inhibit its proliferation, which decreases the quantity of ECs, leading to the damage of the vascular endothelial barrier. At this time, circulating leukocytes and LDL accumulate under a damaged endothelium, leading to the development of AS (126). After *P. gingivalis* infection, pro-apoptotic proteins Bcl−2−associated X protein and CAS3-CL (127) expressed by ECs increased, while the anti-apoptotic protein Bcl−2 decreased significantly (128). *P. gingivalis* gingipains can also induce EC apoptosis, mainly through inducing the cleavage of topoisomerase I (Topo I) and Poly (ADP-ribose) polymerase (PARP), which may regulate the process of cell death to a certain extent (122). The death of apoptotic ECs usually occurs through the stimulation of activated caspase (129). *P. gingivalis* induces EC apoptosis by activating the caspase-8 death receptor and the caspase-9 mitochondrial-dependent apoptosis pathway as well as activating the DNA fragmentation induced by caspase-3 (130). *P. gingivalis* can also induce ER stress with the expression of several growth arrest- and DNA damage-inducible gene 153, glucose-regulated protein 78, and caspase-12, thereby promoting EC apoptosis (130). In addition, several pro-atherosclerotic factors, such as modified lipoproteins and tumor necrosis factor-alpha (TNF-α), affect the *P. gingivalis*-induced death of ECs, as well as other cells, and promote the compound of necrotic cores (107, 131). NLRP3 inflammasome-mediated pyroptosis has been identified as a potential cause of EC death (132), and the production of ROS in ECs with *P. gingivalis* infection may activate the NLRP3 inflammasome. The induction effect of *P. gingivalis* and its LPS on EndMT has been noted in recent studies (111). After the intervention of *P. gingivalis*, the typical paving stone-like ECs become polygonal fibroblast-like cells with enhanced migratory phenotype and an increase happening in long- and spindle-shaped cells, which were suppressed after the use of TLRs–NF-κB pathway inhibitors (127). The expression of EndMT-related proteins have been also changed to cluster of differentiation 31, as the endothelial-specific markers that were downregulated with VE-cadherin were reduced, and α-smooth muscle actin (α-SMA) related with mesenchymal transition was upregulated, which may be mediated by p38, extracellular signal-regulated kinase 1/2 (Erk1/2), and p65 (111). The proliferation of ECs is indispensable in repairing the shedding area of ECs and maintaining the integrity of the endothelium, but it can be significantly inhibited by *P. gingivalis* (127, 130). *P. gingivalis* and its OMVs significantly inhibit the proliferation and growth of ECs (133) and suppressed capillary tube formation by ECs, in which NF-κB signaling played a critical role.

### *P. gingivalis* Survives in Endothelium Leading to Constant Stimulation

In the AS initial stage, *P. gingivalis* can introduce ECs to internalize it and begin autophagy, which was utilized in transporting bacteria or/and toxins. In *in vitro* experiments, *P. gingivalis* invaded ECs through ICAM-1-mediated endocytosis (134). After that, EC autophagy induced by *P. gingivalis* provides a replicative niche where bacteria survive and replicate while suppressing apoptosis (135). The conclusion that the increased endoplasmic reticulum-associated protein Beclin-1 and microtubule-associated protein light chain 3-II can draw is that the endoplasmic reticulum stress induced by *P. gingivalis* enhances autophagy (130, 136). *P. gingivalis* interacts with ECs through a variety of adhesin, including FimA (137) and hemagglutinin B (HagB) (138), and is subsequently transformed into phagosomes through the internalization of lipid rafts (139). After invading ECs, *P. gingivalis* is swallowed by phagosomes to form early autophagosomes. Thereafter, delaying autophagosome–lysosome fusion or redirecting autophagosomes prevent the formation of autolysates to avoid being destroyed (140). In general, *P. gingivalis* directly forms late autophagosomes from early autophagosomes to survive and persist in ECs, but the mechanism remains unclear. Interestingly, scholars have explained that p38 mitogen-activated protein (MAP) kinase in monocytes can be activated by *P. gingivalis*, but ECs do not obey this law, which was shown by the fact that *P. gingivalis* and its LPS have no activation effect on p38 or ERK MAP kinase (141). On the contrary, the effect of MAP kinase in ECs is interfered by *P. gingivalis* LPS in the progress of modulating host defenses, which may also be helpful for *P. gingivalis* survival and replication in ECs and lead to the development of AS.

In general, after *P. gingivalis* reaches the endothelium, it is internalized by ECs and induces autophagy to preserve its virulence (135). Hereafter, *P. gingivalis* activates oxidative stress of ECs, which release a large amount of ROS and inflammatory factors, amplifying the inflammatory response through TLRs–NF-κB and NLRP3 pathways (22, 102). Apart from this, *P. gingivalis* can also increase the permeability of the endothelium by destroying the connections between ECs directly (120). Last but not least, *P. gingivalis* destroys the completeness of the endothelium through the promotion of apoptosis and the inhibition of proliferation in ECs (130). *P. gingivalis* causes endothelial dysfunction and damage in many ways, which all promote the occurrence and development of AS. This also helps us understand that microbial infection plays a role in the pathological development of AS so as to find more effective treatments (**Table 1** and **Figure 3**).

**Table 1:** The effect of *P. gingivalis* on endothelial cells.

Stimulus component	Signal pathway	Target	Outcome	References
LPS	TLRs–NF-κB	p65	Oxidative stress	(22)
Unknown	GSK-3β/BH4/eNOS/Nrf2	DHFR, GCH-1, NrF2	Oxidative stress	(107, 108)
Gingipains	Caspase pathway	Caspase-3,8,9,12	Apoptosis	(130, 142)
Lipopolysaccharide (LPS)	TLRs–NF-κB	p38, Erk1/2, p65	EndMT	(111, 127)
Unknown	TLRs–NF-κB	Unknown	Proliferation	(127)
Rab5, MPR	Unknown	Unknown	Autophagy	(140)
FimA, LPS, gingipains, OMVs	NF-κB	MCP-1, Rac1, PI3K	Adhesion	(114–116)

## The Influence of *P. gingivalis* on Vascular Smooth Muscle Cells

### *P. gingivalis* Promotes the Proliferation and Migration of VSMCs

VSMC proliferation is suggested to contribute to diffuse intimal thickening in AS (143). VSMCs infected with *P. gingivalis* have shown an increasing trend of cell growth and a significant transition from the contractile phenotype to proliferative phenotype. *P. gingivalis* and its gingipains upregulate osteopontin (OPN), *SMemb*, and *S100A9* expression, which were contrary to α-SMA and have an important role in cellular proliferation (144, 145). Gingipains may trigger the proliferation of VSMCs by cleaving plasma proteins at the lysine and arginine residues (145). This process may be mainly regulated by the transforming growth factor-beta (TGF-beta)/Notch pathway (146). *P. gingivalis* mediates the upregulation of connective tissue growth factor [small body size (a *Caenorhabditis elegans*) mothers against decapentaplegic (a *Drosophila* protein family)-3 (SMAD3)], which are signaling molecules of the TGF family (147). *Hairy/enhancer−of−split related with YRPW motif 1* (*HEY1*) and *Notch1*, as two key genes of the Notch pathway (148), were upregulated in VSMCs infected with *P. gingivalis*. Moreover, a multi-center cohort study in Japan showed that the intima-media thickness was significantly decreased in patients after control of periodontal infection by periodontal treatment (149). This implies that inhibiting *P. gingivalis* infection can decrease the thickness of plaques to reduce the risk of rupture, which is related to inhibition of the proliferation of VSMCs.

The migration of VSMCs from the middle layer to the inner layer of the blood vessel is a key event of the progression of AS (150). *P. gingivalis* gingipains enhanced the migration ability of VSMCs by upregulating angiopoietins 2 (Angpt2) and ETS proto-oncogene 1 (ETS1) while inhibiting Angpt1. ETS1 is the transcription factor of Angpt2, which is critical for *P. gingivalis* to induce Angpt2 (151). Angiopoietins (Angpt1, Angpt2, *etc.*) regulate vascular maturation, stability, and remodeling by the Tie2 receptor signaling pathway (152), in which Angpt2, particularly, enhanced VSMCs to migrate but had no influence on its proliferation. In addition, after the invasion from *P. gingivalis* to VSMCs, its LPS could significantly reduce the expression and activity of tissue factor inhibitor, thereby inducing the migration as well as the proliferation of VSMCs through which atherosclerotic plaques have been promoted (153). Unexpectedly, for *C. pneumoniae*, as another Gram-negative pathogen associated with AS, its infection was found to promote VSMC migration *via* c-Fos/IL-17C signaling (154). Despite the same outcome from both of them, they promote plaque progression in different pathways.

### *P. gingivalis* Promotes the Calcification of VSMCs

Vascular calcification, described as excessive deposition of calcium-containing phosphate, is one of the signs of AS (83), which might ultimately lead to the hardening of blood vessels and reduction in elasticity (155). *P. gingivalis* can induce the calcification of VSMCs and promote vascular calcification, which is also induced by LPS and OMVs. The OMVs of *P. gingivalis* promoted the calcification of VSMCs, along with the involvement of the ERK1/2- Runt-related transcription factor 2 (RUNX2) pathway (156), in a concentration-dependent manner and regulate the process of VSMC osteogenic differentiation and mineralization (157). The key regulator of this progress is Runx2, regulated by the ERK signaling pathway and involved in osteogenic transcription (158). Bone morphogenetic protein 4 was upregulated and mediated by TLR4 and ERK1/2-p38 pathway, and ultimately it promoted vascular calcification in VSMC from one suffering from *P. gingivalis* infection (159). During vascular calcification, VSMCs change to an osteoblast-like phenotype, which is an important step in mediating vascular media calcification (160), and *P. gingivalis* LPS significantly promoted the upregulation of osteogenic genes [such as *alkaline phosphatase* (ALP), *core-binding factor*, *alpha 1*, *bone sialoprotein*, and *OPN*] (161). Moreover, apoptosis of VSMCs, accompanied by considerable matrix vesicles with the bound calcifying membrane released (162), was increased by *P. gingivalis* in inorganic phosphate-induced VSMCs, in which the Gas6/Axl/Akt survival pathway was inhibited (163). In addition, TNF-α and IL-1β, as pro-inflammatory cytokines, can upregulate ALP and RUNX2 in VSMCs, contributing to vascular calcification (164). Therefore, *P. gingivalis* may promote vascular calcification through its structure or secreted substances and, alternatively, through the secretions of VSMCs after infection. Matrix-gla protein (MGP), an effective inhibitor of vascular calcification, and lack of MGP will increase the risk of AS (165, 166). However, the relationship between MGP and *P. gingivalis* is still unclear, which also suggests our next research direction, that is, whether MGP can be used to alleviate vascular calcification caused by *P. gingivalis.*

### *P. gingivalis* Promotes VSMCs to Engulf Lipids to Form Foam Cells

As the most indispensable cell in AS lesions, VSMCs contribute to the proportion of more than half of the foam cells in the lesion area (167). In the process of that, the aggregation of LDL and its oxidative modification product oxLDL as well as the immune complexes they induce, such as b2glycoprotein I (b2GPI), anti-b2GPI, plays a key role in promoting the formation of foam cells (168). Recent studies have shown that the lipid uptake pathways of VSMCs include SR-AI/II (class A), CD36 (class B), LOX-1 (class E), and SR-PSOX/CXCL16 (class G) (169). In addition, the presence of macrophages also promotes the transformation of SMCs into foam cells (170). At present, the research on the effect of *P. gingivalis* on VSMCs is not sufficient and in depth. VSMCs may serve as deposits of lipids from the insudating lipoproteins and become foam cells easily with *P. gingivalis*, but the *in vivo* mechanisms remain incompletely understood (85). According to our research, *P. gingivalis* promotes the accumulation and oxidation of lipids under the endothelium, and *P. gingivalis* promotes the chemotaxis of macrophages. Therefore, we speculate that *P. gingivalis* can promote the uptake of lipids by VSMCs and form foam cells through these pathways (**Table 2**).

**Table 2:** The effect of *P. gingivalis* on vascular smooth muscle cells.

Stimulus component	Signal pathway	Target	Outcome	References
Unknown	TGF-beta/Notch	SMAD3, GO categories, bHLH (HEY1, *etc.*)	Proliferation	(146)
Unknown	Notch1 signaling cascade	HES1, HEY1	Proliferation	(146)
Gingipains	Unknown	S100A9	Proliferation and transformation	(145)
Gingipains	Unknown	Angpt2, ETS1, Angpt1	Migration	(151)
OMVs	ERK1/2-RUNX2	Runx2	Calcification	(156)
LPS	Unknown	ALP, Cbfa1, BSP, OPN	Calcification	(161)

## The Influence of *P. gingivalis* on Macrophages

### *P. gingivalis* Achieves Immune Evasion Through Macrophages

*P. gingivalis* achieves immune evasion through internalization by macrophages, which may result in the preservation of *P. gingivalis* virulence and chronic infection during AS. The uptake of *P. gingivalis* by macrophages hinges on complement receptor type 3 [CR3 (CD11b/CD18)] and TLR2 (171). CR3 is a β2 integrin, which can recognize sort of structurally and morphologically unrelated molecules from a pathogen or a host, such as intercellular adhesion molecules, fibrinogen, and so on (172). *P. gingivalis* selected the TLR2 pre-pathway to bind CR3 and entered the cell (173). The intracellular *P. gingivalis* stimulates TLR2 through its surface fimbriae and activates the signal pathway from the inside out to induce a distinct conformation of CR3 with high affinity. This pathway can be mediated by Rac1/PI3K, and it requires fimbriae to bind CD14 to promote fimbria–TLR2 interaction (117, 174). What is more, CR3 is utilized as a relatively safe portal of entry by *P. gingivalis*, and its fimbriae additionally inhibited the production of IL-12 (p70) with biological activity by interacting with CR3 on the surface of macrophages (171, 174, 175), which support that *P. gingivalis* achieves evasion immune clearance.

### *P. gingivalis* Promotes the Inflammatory Response of Macrophages

Overwhelming experimental and clinical evidence suggest that AS is a chronic inflammatory disease (176). *P. gingivalis* triggers the inflammatory response of macrophages, thereby promoting different stages of AS. *P. gingivalis* fimbriae and OMVs stimulated monocytes and macrophages to secret pro-inflammatory cytokines—for instance, IL-1β, IL-18, TNF-α, and NLRP3 inflammasome activation (49, 177). Under the action of TNF-α and IL-6, the activation and antigen presentation ability of macrophages are embellished, and immunity is also modulated by different mechanisms (178). Furthermore, the TNF-α released by macrophages can promote EndMT in ECs (111), which can be promoted by *P. gingivalis*. Therefore, it is possible for *P. gingivalis* to induce EndMT of ECs by promoting the secretion of TNF-α from macrophages (111). *P. gingivalis* can easily interact with activated CR3, activating the outside-in signaling pathway, and lessen IL-12 due to ERK1/2, thereby inhibiting the production of biologically active (p70) IL-12 (174, 179), which mediates immune clearance (180). We can understand from studies that *P. gingivalis* LPS and gingipains activated the macrophage NLRP3 inflammasomes and then produced powerful inflammatory cell factor IL-1β with the activation of NF-κB signaling (181), which makes M1-Mϕ secrete TNF-α and M2-Mϕ secrete IL-10, along with chemotactic chemokines like knuckle cracking (regulated upon activation, normal T cell expressed and secreted)/CC chemokine ligand 5, eotaxin, and IL-10 from polarized macrophages (182). In particular, *P. gingivalis* OMVs were also suggested to minimize anti-inflammatory IL-10 secretion (183), and NLRP12 is upregulated, which downregulated TNF-α production and iNOS expression in macrophages infected with *P. gingivalis* (184). Moreover, *P. gingivalis* gingipains reduced the expression of CD14 in macrophage to reduce macrophage interactions with apoptotic cells, which could curb TNF-α-induced expression by *P. gingivalis* LPS (185).

### *P. gingivalis* Promotes Macrophages to Form Foam Cells

**Figure 4:**
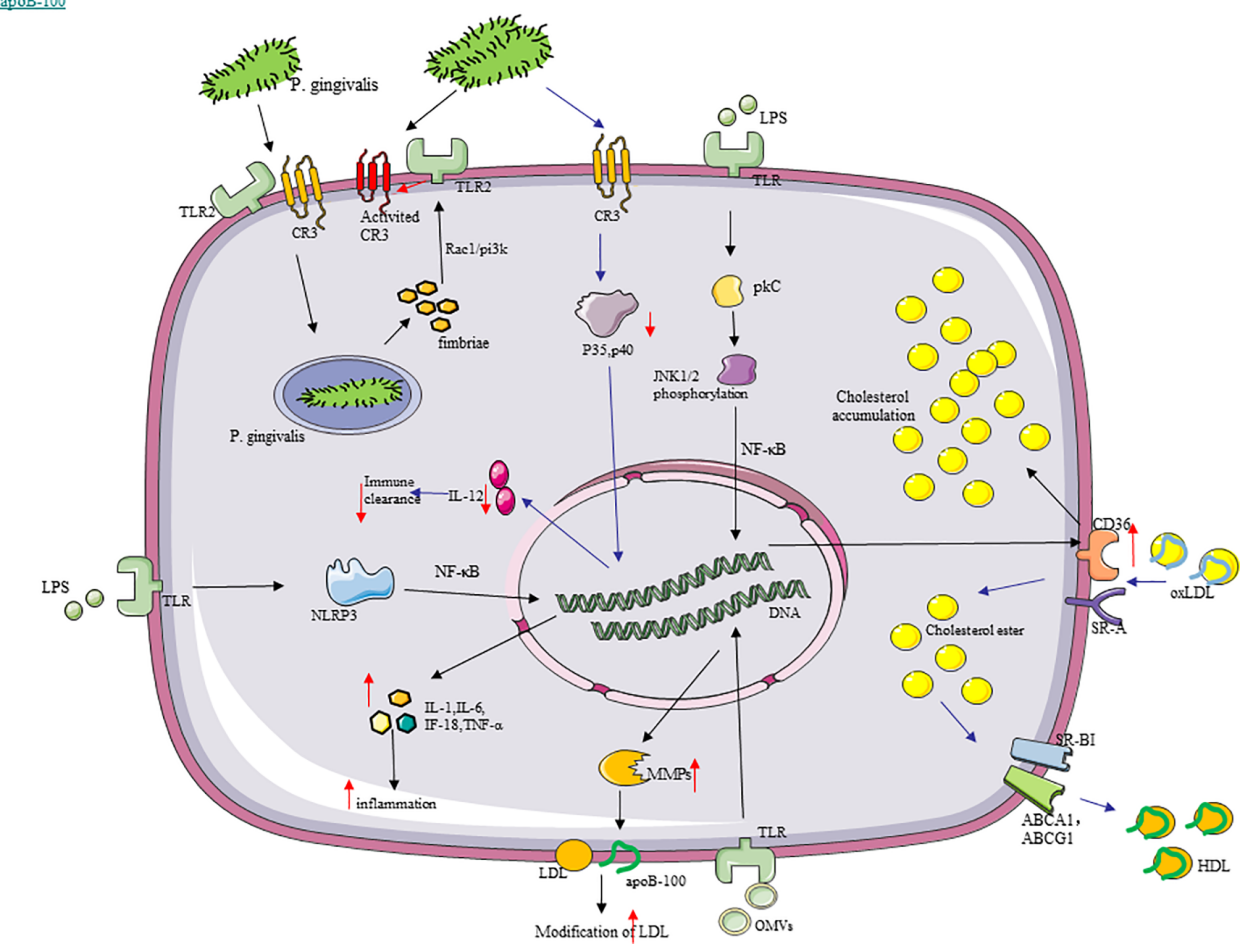
Molecular cascades activated by *P. gingivalis* in macrophages. (1) *P. gingivalis* recognizes TLR2, binds to CR3, and enters macrophages. The intracellular *P. gingivalis* stimulates TLR2 through its surface fimbriae, thereby inducing the high-affinity conformation of CR3, which is conducive to the uptake of more *P. gingivalis* by macrophages. (2) SR-A and CD36, as the clearance receptors of macrophages, mediate the internalization of oxLDL, thereby promoting the accumulation of intracellular cholesterol. In contrast, SR-BI and ABCA1/ABCG1 are responsible for cholesterol efflux. *P. gingivalis* lipopolysaccharide increased the expression of CD36 through the c-Jun-AP-1 pathway and promoted cholesterol accumulation in macrophages. (3) *P. gingivalis* outer membrane vesicles stimulated the macrophages to produce matrix metalloproteinases capable of cleaving the apoB-100 of low-density lipoprotein (LDL) particles to increase the modification of LDL. (4) *P. gingivalis* promoted the production of pro-inflammatory cytokines, like IL-1, IL-18, IL-6, and TNF-α in macrophages, with the activation of NLRP3 inflammasomes.

Foam cells are critically important to the development and progression of AS, and *P. gingivalis* promotes macrophages to form foam cells. With LDL, low-concentration *P. gingivalis* OMVs also induce foam cell formation, which is in a dose-dependent manner (186). Firstly, *P. gingivalis* and OMVs can provoke LDL modification (187), which is vital in AS by acting on multiple cells (188), and can be easily transported to macrophages to form foam cells. *P. gingivalis* OMVs stimulated the macrophages to produce matrix metalloproteinases (MMP), and a few types of them were able to cut apolipoprotein B-100 (apoB-100) from LDL particles and then lead to the aggregation and modification of LDL (189, 190). *P. gingivalis* induced the conversion of macrophage metabolism from oxidative phosphorylation to glycolysis, which enhances the release of lactic acid, reduces mitochondrial oxygen consumption, and increases ROS (191), so that modification of LDL may be increased by ROS in macrophages with *P. gingivalis*. Secondly, *P. gingivalis* fimbriae significantly promotes the uptake of LDL by macrophages to form foam cells (187), with *P. gingivalis* LPS enhancing lipid accumulation in macrophages and reducing cholesterol efflux (192). The clearance receptors of macrophages, such as the class A clearance receptor (SR-A) and CD36, mediate the internalization of oxLDL, thereby promoting the accumulation of intracellular cholesterol (193). In contrast, reverse cholesterol transporters including scavenger receptor class B type I (SR-BI) and adenosine-binding cassette transporters A1 and G1 (ABCA1/ABCG1) are responsible for cholesterol efflux (194). *P. gingivalis* LPS reduced ABCA1 in macrophages but increased CD36 through the c-Jun-AP-1 pathway, while it did not affect SR-A, SR-BI, and ABCG1 (195). These outcomes are partially associated with the activation of protein kinase C and c-Jun N-terminal kinase 1/2 phosphorylation, which promotes NF-κB to activate (110). At the same time, *P. gingivalis* LPS reduces the stability of ABCA1 protein by increasing calpain activity (195). Moreover, the TLR signal transduction in macrophages is mainly composed of MyD88 and Toll/IL-1R domain-containing adaptor-inducing IFN-β, and it exercises function in foam cells caused by *P. gingivalis* (196) (**Figure 4**).

Hence, chronic inflammation caused by *P. gingivalis* might gradually worsen in this way. In addition, macrophages can generate inducible proteases under the action of *P. gingivalis*, such as MMP, which can crack cytokine precursors, growth factors, cytokine receptors, and cell adhesion molecules (122). In summary, *P. ging*ivalis has a considerable immunomodulatory impact and can act on monocytes and macrophages, producing various inflammatory mediators and enzymes through these pathways to promote inflammation and tissue damage in the process of AS (**Figure 4**).

## The Influence of *P. gingivalis* on T Cells

### *P. gingivalis* Causes Th17/Treg Imbalance

**Figure 5:**
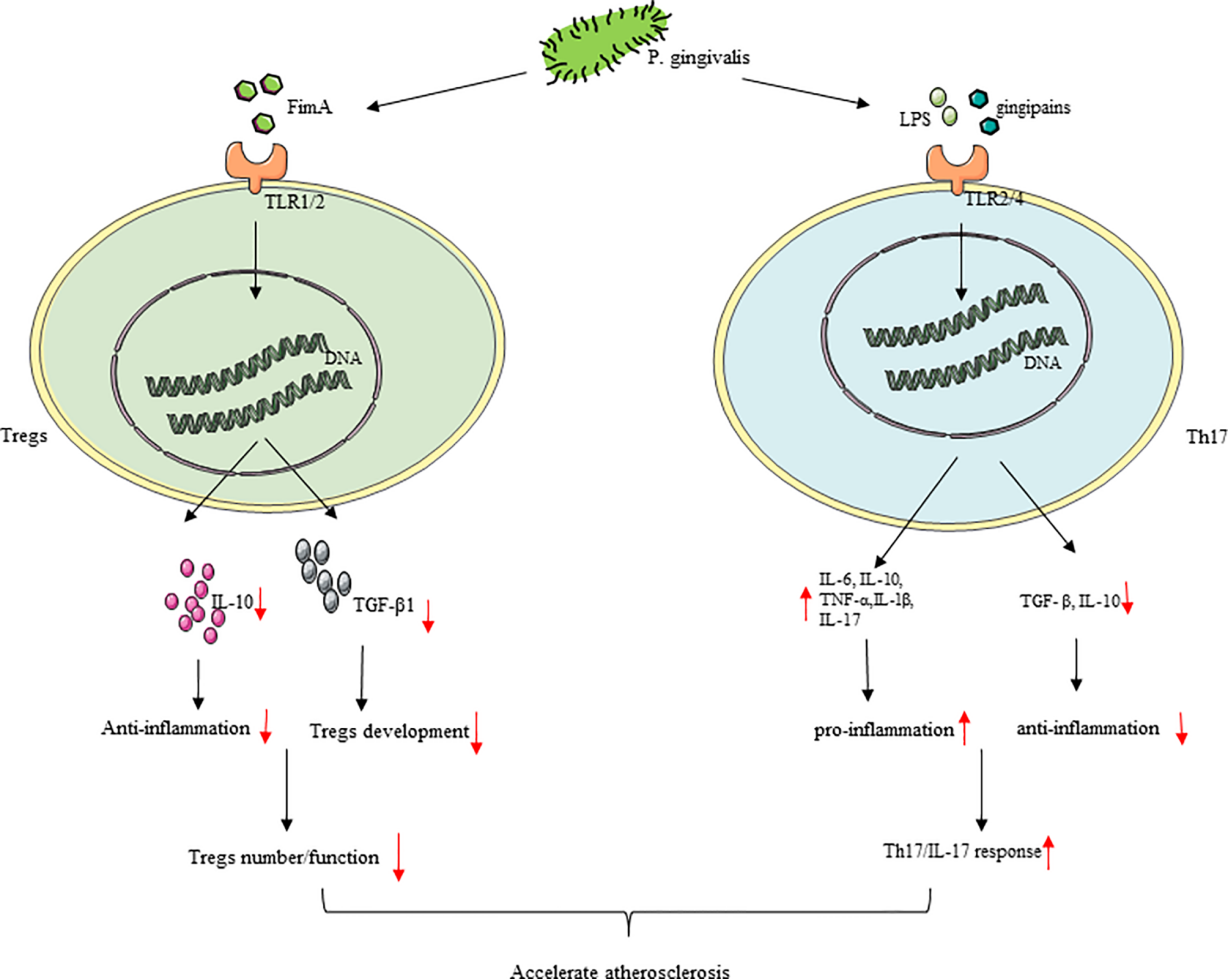
*P. gingivalis* infection cause Th17/Treg imbalance. (1) In the process of atherosclerosis, *P. gingivalis* infection increased the number and response of Th17, inhibited Tregs with regulatory effects, and cause Th17/Treg imbalance. (2) *P. gingivalis* reduced the number and inhibited the regulatory function of Tregs with the downregulation of IL-10 and TGF-β1. *P. gingivalis* promoted a Th17/IL-17 response resulting in increased TNF-α, IL-1β, IL-6, and IL-17 production by T cells, which may be mediated by TLR2/TLR4 signaling.

The immune balance between Th cells and Tregs has an important regulatory role in AS (71). With the development of AS, the number and response of Th17 will increase accordingly with *P. gingivalis* infection, reducing the number and inhibiting the regulatory function of Tregs and causing Th17/Treg imbalance, which may lead to plaque instability (197, 198). *P. gingivalis* and its LPS and gingipains can activate monocytes, promote a Th17/IL-17 response, and then make TNF-α, IL-1β, IL-6, and IL-17 increase, mediating by TLR2/TLR4 signaling and inducing atherosclerotic plaque formation through an inflammatory response (199, 200). The Th17-related genes like *IL-6*, *retinoid-related orphan receptor-gammat* (*RORγt*), and STAT3 were elevated, with *TGF- β* and *IL-10* decreasing in *P. gingivalis*-challenged mice (201). *P. gingivalis* used gingipain to highly specifically induce Th17 cells, by which IL-6 signaling was blocked (202). Furthermore, *P. gingivalis* infection promotes IL-6 to secret in DCs, and increased IL-6 may be good for Th17 cell proliferation and may inhibit the production and effect of Tregs (203). In general, pro-inflammatory Th17 cell responses were strengthened by *P. gingivalis*, thereby accelerating AS. Tregs can inhibit effector T cell proliferation and the production of cytokine (mainly Th1 and Th17 lymphocytes) and are vital in maintaining the homeostasis of the immune-inflammatory response of the host (204). *P. gingivalis* infection reduces the number and inhibits the regulatory function of Tregs. Compared with people in healthy conditions, AS patients with *P. gingivalis* have fewer Tregs (198). Tregs themselves can come into contact with other effector cells and can also secrete anti-inflammatory IL-10 and TGF-β1, thereby directly or indirectly inhibiting inflammation (197, 205). TGF-β1 has contributed much to the development of Tregs (206). IL-10 is a cytokine that contributed a lot to anti-inflammatory effects (207). Experiments have shown that *P. gingivalis* infection reduces IL-10. TLR2/1 signaling is the main mechanism of IL-10 production. Thirdly, the reaction caused by the main surface protein of *P. gingivalis* FimA is also involved among them (48), and the concentration of Treg-related factors like TGF-β1 and FoxP3 was reduced in *P. gingivalis*-positive patients (198). According to reports, there is Th17/Treg imbalance in AS patients, and immune answer induced by T cell is principal in plaque instability (208, 209). As a result, *P. gingivalis* ultimately induces an increasing inflammation reaction in AS plaque and plaque instability by promoting Th17/Treg unevenness (**Figure 5**).

### *P. gingivalis* Inhibits T Cell Answer

*P. gingivalis* can inhibit T cell answer through the suppression of differentiation and activation caused by chemokines, proliferation, and communication in T cells. Firstly, the T cell chemokine interferon-inducible protein 10 or CXC motif chemokine 10, which comes from neutrophils and monocytes, were not influenced by *P. gingivalis*, which makes a T cell respond weakly and achieve local immune evasion (210). Secondly, the CD4 and CD8 proteins on human T cells can be destroyed by *P. gingivalis*, thereby inhibiting the activation of T cells. It is also through this mechanism that *P. gingivalis* can protect itself from the immune system. The establishment and proliferation of the bacteria in the host is achieved (211). *P. gingivalis* HSP60 can have different effects on T cell polarization by different mechanisms and then make atherosclerotic occur or not (212). Last but not least, *P. gingivalis* inhibits the expression of IL-2 that promotes the proliferation and communication of T cells (101). The activity of NF-kB and AP-1 is downregulated by *P. gingivalis* and its Rgp protease, so IL-2 cannot be transcribed and expressed (213), and then IL-2 cannot stably accumulate in T cells, which resulted in T cells without energy (214). This weakens the inflammatory response, which is connected with T- and B-cell activation, and subsequently IFN-γ from T cells (101). We have a clearer understanding of how *P. gingivalis* can prevent itself from being cleared by the immune system by inhibiting T cell response and thus surviving and persisting in AS lesions to promote AS development (**Figure 5**).

## The Influence of *P. gingivalis* on DCs

DCs can promote lipid accumulation, promote inflammation, and destroy plaque stability during various stages of AS (99, 215). It is worth noting that there are many connections between DCs and *P. gingivalis*. DCs can be used as carriers to transport pathogenic bacteria such as *P. gingivalis* from the oral cavity with serologic exposure through the bloodstream to reach the arteries in the pre-AS lesions (216–218). The minor fimbria of *P. gingivalis* binds with a cell adhesion molecule on DCs called CD209 to enable it to escape immune surveillance (219). *P. gingivalis* promotes both its own survival and the survival of its host DCs through manipulating dendritic cell signaling to perturb both autophagy and apoptosis, in which activation of the Akt/mTOR axis was linked. There was also the induction of the anti-apoptotic protein Bcl2 and decrease of caspase-3 cleavage and pro-apoptotic proteins Bax and Bim in this progress (220).

## Conclusion

In recent years, the promotion of *P. gingivalis* in the pathological process of AS has received more attention. *P. gingivalis* has the capability of leading to arterial endothelial dysfunction, inducing foam cell formation, and making vascular smooth muscle cells proliferate and calcify, causing T helper cells and Tregs imbalance. Accompanied with the progression of endothelial activation, lipid accumulation, plaque formation, and rupture, *P. gingivalis* eventually aggravates the process of AS. Here we summarized and provided several of the latest research findings on the effects of *P. gingivalis* on AS-related cells as well as the underlying mechanisms, which may help to provide new insights on the targets for the effective treatment and prevention of AS.

## Author Contributions

JZ and MX performed the original draft preparation and revision, created the tables and figures, and were the major contributors in writing the manuscript. XH, GC, and YY made suggestions to the writing of the manuscript and revisions to tables and figures. XL and GF participated in conceptualization and methodology. GC supervised the work and acquired funding. All authors contributed to the article and approved the submitted version.

## Funding

This work was supported by the National Natural Science Foundation of China for Key Program Projects (no. 82030070), the National Natural Science Foundation of China for Distinguished Young Scholars (no. 31725011), Hubei Provincial Natural Science Fund for Creative Research Groups (2020CFA014), National Natural Science Foundation of China for Young Scientists (no. 82101025), and Key Supporting Program by the Health Commission of Hubei Province (WJ2019C001).

## Conflict of Interest

The authors declare that the research was conducted in the absence of any commercial or financial relationships that could be construed as a potential conflict of interest.

## Publisher’s Note

All claims expressed in this article are solely those of the authors and do not necessarily represent those of their affiliated organizations, or those of the publisher, the editors and the reviewers. Any product that may be evaluated in this article, or claim that may be made by its manufacturer, is not guaranteed or endorsed by the publisher.

## Glossary

**Table:**

ABCA1/ABCG1	adenosine-binding cassette transporters A1 and G1
ALP	alkaline phosphatase
Angpt2	angiopoietins 2
AP-1	activator protein 1
α-SMA	α-smooth muscle actin
apoB-100	apolipoprotein B-100
AS	atherosclerosis
Axl	a TAM (TYRO3
AXL	and MERTK) family receptor tyrosine kinase
b2GPI	b2glycoprotein I
BAX	BCL2-related X
BH4	tetrahydrobiopterin
*C.pneumoniae*	*Chlamydia pneumoniae*
CR3	complement receptor type 3
CVD	cardiovascular disease
DCs	dendritic cells
DHFR	dihydrofolatereductase
ECM	extracellular matrix
ECs	endothelial cells
EndMT	endothelial cell–mesenchymal transition
eNOS	endothelial nitric oxide synthase
ERK	extracellular signal-regulated kinase
Erk1/2	extracellular signal-regulated kinase 1/2
ETS1	ETS proto-oncogene 1
FoxP3	lineage-defining transcription factor of CD4+ CD25+ regulatory T cells
GAS6	growth arrest-specific 6
GCH-1	GTP cyclohydrolase 1
GSK-3	glycogen synthase kinase-3
Hag	hemagglutinin
HagB	hemagglutinin B
HEY1	hairy/enhancer−of−split related with YRPW motif 1
HSP	heat-shock proteins
ICAM-1	intercellular adhesion molecule-1
IFN-γ	interferon-gamma
IL	interleukin
iNOS	inducible nitric oxide synthase
LDL	low-density lipoprotein
LPS	lipopolysaccharides
MAP	mitogen-activated protein
MCP-1	monocyte chemoattractant protein-1
MGP	matrix-gla protein
I	myocardial infarction
MIF	migration inhibitory factor
MMP	matrix metalloproteinases
NLRP3	nucleotide-binding domain leucine-rich repeat (NLR) and pyrin domain containing receptor 3
Nrf2	nuclear factor erythroid-derived 2-like 2
OMVs	outer membrane vesicles
OPN	osteopontin
oxLDL	oxidized low-density lipoprotein
PARP	poly(ADP-ribose) polymerase
PECAM-1	platelet endothelial cell adhesion molecule-1
*P. gingivalis*	*Porphyromonas gingivalis*
PI3K	phosphatidylinositol 3-kinase
PIT	pathological intimal thickening
Rac1	Ras-related C3 botulinum toxin substrate 1
Rgp	arginine–gingipain
RORγt	retinoid-related orphan receptor-gammat
ROS	reactive oxygen species
Runx2	Runt-related transcription factor 2
SMAD3	[small body size (a *C. elegans* protein) mothers against decapentaplegic (a *Drosophila* protein family)]-3
SMemb	nonmuscle myosin heavy chain B
SR-A	class A clearance receptor
SR-BI	scavenger receptor class B type I
STAT3	transcription 3
TGF-β	transforming growth factor-beta
TH1	T helper cells 1
TLRs	Toll-like receptors
TNF-α	tumor necrosis factor alpha
Topo I	topoisomerase I
Tregs	regulatory T cells
VSMCs	vascular smooth muscle cells
α-SMA	α-smooth muscle actin
